# Homœopathic Grammar

**Published:** 1886-03

**Authors:** 


### EDITORIAL NOTES.

### HOMŒOPATHIC GRAMMAR.

During the "rib" controversy which was carried on in the *Statesman* recently, between the city physician of Austin, and the editor of the homœopathic journal (?) the following choice sentence occurred, written by the latter. In that controversy, it will be remembered, this homœopathic surgeon (?) was accused (on his own statement, as charged) of having removed the twelfth rib of a man who was shot in the spinal column, in search of the ball, which it was alleged the h. s. had said he would "find lodged in the abdomen." This article has nothing to do with the case, but is intended only to show with what facility this surgeon-editor—President of the State Association of forty homœopaths—uses the king's English :

"We have extracted no ribs that we recollect of. It does not belong to our 'pathy' no more than to theirs to make such egregious blunders as that would have been."

"Recollect of" is decidedly weak ; but "does not no more," must be a high potency attenuation. When a man who is unacquainted with the simplest rule in grammar—that two negatives destroy each other, and are equivalent to an affirmative" —mounts the tripod, and essays to edit a journal, "an eligible opportunity," as Mr. Pecksniff would say, is afforded the public for witnessing the "ears" protruding from the lion skin.

Apropos: Our Dr. Merryman saw the above, and, like the "late lamented," he said it "reminded him of the anecdote of

